# Improving the Quality of Life of Gynecological Patients with Surgery: A Cross-Sectional Study Based on Questionnaire Surveys

**DOI:** 10.3390/medicina61091706

**Published:** 2025-09-19

**Authors:** Mariola Disterheft-Komisarek, Maciej Wilczak, Katarzyna Wszołek, Karolina Chmaj-Wierzchowska

**Affiliations:** Department of Maternal and Child Health and Minimally Invasive Surgery, Poznan University of Medical Sciences, 60-535 Poznan, Poland; mdisterheftkomisarek@gpsk.ump.edu.pl (M.D.-K.); mwil@ump.edu.pl (M.W.)

**Keywords:** minimally invasive surgery, gynecology, quality of life, laparoscopy, patient-reported outcomes

## Abstract

*Background and Objectives*: Gynecological conditions requiring surgical intervention have a profound impact on women’s physical, psychological, and social well-being. Minimally invasive procedures are increasingly preferred due to their benefits in reducing recovery time and improving patient outcomes. This study aimed to assess the changes in quality of life (QoL) among women undergoing surgical treatments for gynecological diseases. *Materials and Methods*: A cross-sectional study was conducted among 150 women hospitalized at the Minimally Invasive Pelvic Surgery Center. The participants completed two surveys: one prior to surgery and the other one month postoperatively. A self-developed questionnaire was used, tailored to the clinical and psychosocial context of the study population. Statistical analyses included the Wilcoxon signed-rank test for paired comparisons and the Chi-square test for categorical variables. A significance level of *p* < 0.05 was applied. *Results*: After surgery, 92% of women reported improved daily functioning, and 90% experienced a better overall quality of life. The average level of limitations decreased significantly from 40% to 16% (*z* = 11.24, *p* < 0.001). Higher BMI was associated with greater limitations both before and after surgery (*r* = 0.31 and *r* = 0.24, respectively; both *p* < 0.001). *Conclusions*: Women with uterine fibroids showed less improvement in several QoL domains compared to those with other conditions. Psychological factors, such as anxiety and long-term distress, play an important role in both pre- and postoperative well-being. Optimizing postoperative quality of life requires consideration of both the surgical method and individual psychosocial circumstances.

## 1. Introduction

Modern operative gynecology advocates for implementing minimally invasive and rapid treatment above all [1,2,3]. The ability to quickly diagnose and take necessary action contributes to the patient’s experience, as they often “don’t have that much time” to evaluate their situation negatively. Therefore, the sooner treatment is applied, the greater the chance that the patient will, over time, forget what happened and return to normal life after the procedure and recovery [4].

Any patient qualifying for a procedure within operative gynecology faces various types of discomfort over an extended period that significantly impair their comfort and quality of life. Therefore, the opportunity to undergo treatment and a procedure that can greatly reduce their discomfort positively impacts how they view their future life. Patient quality of life, both after and even before surgery, is influenced by the treatment itself and the planned surgical intervention. It is important to note that the diagnostic process and the diagnosis of the disease affect the patient’s mood, often leading to lowered spirits or even depression [5,6,7]. Any side effects associated with the treatment also significantly shape the patient’s perception of their situation within the treatment process [8].

Patients requiring surgical treatment represent a particularly demanding group requiring a specialized approach characterized by understanding and empathy for the emotions they experience [9]. According to several studies, the extent and manner in which information about the diagnosis and related treatment methods is communicated can enhance cooperation with the patient. This improved cooperation is reflected in better collaboration with the medical team, increased satisfaction with care, and a greater sense of control over the situation [6]. For this reason, it is not surprising that these women are eager to obtain all the information that is most important to them regarding the disease necessitating surgical treatment and the actions doctors will take before and after the operation, along with their purposes [4].

It is important to note that one element affecting the assessment of the quality of life of female patients may be the ubiquitous cult of the body in modern culture [10]. The diagnosis of an illness requiring surgery (even laparoscopic methods) can realistically contribute to a decrease in quality of life for these patients [11,12]. This situation can be difficult for those around them to understand, as, in the context of the disease itself, it may not seem so significant; after all, the most important aspect is the health and life of the patient [8]. In conclusion, it can be stated that every patient has the right to feel uncomfortable about their discomfort, have concerns regarding their health and treatment, and demand that all information about their treatment be communicated to them.

This study aims to assess the changes in quality of life among women undergoing minimally invasive surgical treatments for gynecological diseases. The proposed tool allowed us to collect detailed information regarding functional limitations, social roles, and subjective health perceptions pre- and post-intervention.

## 2. Materials and Methods

This study included 150 patients admitted to the Minimally Invasive Pelvic Surgery Center at Heliodor Święcicki Gynecological and Obstetrics Clinical Hospital of the Karol Marcinkowski Medical University in Poznań, Poland, for surgery using minimally invasive techniques.

### 2.1. Procedure of Qualifying Patients for Surgery

The criterion for participation was eligibility for surgery, as determined by a prior gynecological examination. Patients were qualified for the procedure following a gynecological and ultrasound (USG) examination and after completing a medical history.

### 2.2. Data Collecting

This study was conducted from January to June 2022, utilizing two questionnaires to assess the quality of life of female patients before surgery and 1 month after surgery. The study group were assured of complete anonymity and voluntary participation.

Each respondent was required to complete the questionnaire twice—before the surgery and 1 month after the surgery—indicating whether their quality of life had changed. Data from nine women were discarded from the study due to incomplete responses.

For the purpose of this study, we created our own two questionnaires to assess women’s quality of life related to their ailments. The patients completed the first questionnaire before the operation, and the second one was completed 1 month after. The questions were designed to best analyze the topics covered by this study’s thesis.

The first survey included questions about the reason for visiting the Minimally Invasive Pelvic Surgery Center at Heliodor Święcicki Gynecological and Obstetrics Clinical Hospital, Poznan University of Medical Sciences, how the patient learned about the possibility of surgery (How did you hear about the Center for Minimally Invasive Pelvic Surgery? Is the outcome of another treatment outside the Center for Minimally Invasive Pelvic Surgery a consequence of the outcome? What made you choose the Center for Minimally Invasive Pelvic Surgery? Would you recommend the Center for Minimally Invasive Pelvic Surgery to another patient?), the duration and severity of their symptoms, their opinion of the medical and obstetric staff (How would you recommend medical work at the facility? How would you recommend nursing work at the facility?), and their socioeconomic conditions and the impact of the disease (0—non, 1—small, 2—average, 3—big, 4—very big, 5—not applicable) on particular aspects of life (^a^ Working life, ^b^ Social life, ^c^ Playing sports, ^d^ Sexual intercourse, ^e^ Enjoying hobbies, ^f^ Having children, ^g^ Dressing in favorite way, ^h^ Using public transportation, ^i^ Driving a car, ^j^ Having a partner, ^k^ Going out shopping, ^l^ Going for a long walk, ^m^ Keeping body weight within the normal range, ^n^ Carrying out previously intended plans, ^o^ Controlling daily expenses). The second survey focused mainly on comparing quality of life after surgery to that in the period before surgery (poor, moderate, good, very good).

This study used a self-developed questionnaire created specifically for the clinical context of the Minimally Invasive Pelvic Surgery Center. The tool was designed to capture patient experiences before and after surgery, with a focus on functional limitations, emotional state, and quality of life. Although not validated against standardized tools such as SF-36 or EQ-5D, it allowed us to obtain targeted insight into the patient population to which it was administered. The use of a non-validated instrument may limit generalizability and comparability with studies using standardized QoL measures. However, the use of non-validated questionnaires is not a weakness; it is required in these modern times of living online and on social media. This limitation is acknowledged in the discussion and should be considered when interpreting the results.

The second survey focused mainly on comparing the quality of life after surgery with that before surgery. This study was approved by the local Bioethics Committee (No 178/21).

### 2.3. Statistical Analysis

Statistical Analysis: For continuous variables such as quality of life (QoL) scores and limitation levels, the Wilcoxon signed-rank test was used to compare pre- and postoperative results. Group comparisons for non-parametric continuous data were performed using the Mann–Whitney U-test or Kruskal–Wallis test. For categorical data, such as age groups, occupation, and type of diagnosis, associations were examined using the Chi-square (χ^2^) test. Spearman’s rank correlation coefficient was used to evaluate relationships between non-parametric continuous variables. The study group included a heterogeneous population: patients with uterine myomas (23.5%), urinary incontinence (19%), pelvic organ prolapse (14.5%), endometriosis (11%), and other undefined gynecological conditions—benign tumors of the ovaries, e.g., teratoma, mucous, or simple cyst (24%). While this reflects real-world clinical diversity, the large proportion of “other” diagnoses limits the clinical specificity of the results, and conclusions regarding this category should be considered purely exploratory.

For data analysis, we used TIBCO Software Inc. (2017) (Palo Alto, CA, USA), Statistica (data analysis software system, version 13), and Microsoft Excel (version 2019) from Microsoft Office. The Mann–Whitney U-test and Kruskal–Wallis ANOVA test were employed for comparisons between the groups. Pre-treatment and post-treatment results were compared using the Wilcoxon paired-rank order test. The relationship between variables was examined using Spearman’s R-test and Chi^2^ NW (highest reliability). The significance level in all calculations was set at *p* < 0.05.

## 3. Results

### 3.1. Characteristics of the Entire Study Group

The study group consisted of 150 women admitted to the hospital for various reasons: uterine myomas (23.5%), urinary incontinence (19%), lowering (8%) and prolapse (14.5%) of the reproductive organ, endometriosis (11%), and other gynecological conditions (24%) requiring surgical treatment. The characteristics of the entire study group are shown in Table 1.

The anthropometric measurements of the respondents, including height and weight, as well as the BMI determined based on these measurements, are shown in Table 2.

This study observed significant differences between the respondents’ ages and the type of condition. Women aged 36–50 years were significantly more likely to have uterine myomas, while women over 51 years of age were more likely to experience urinary incontinence. The youngest respondents were significantly more likely to visit the center for endometriosis and other conditions (*χ*^2^ = 163.98; *p* < 0.001). Women with excessive body weight were significantly more likely to have urinary incontinence and lowering or prolapse of the reproductive organ, while women of normal weight were significantly more likely to report to the center for other conditions (*χ*^2^ = 71.97; *p* < 0.001). These relationships are shown in Table 3.

The time from the appearance of the first symptoms to diagnosis among female respondents ranged from 2 months to 20 years, with an average of 5 years (6.47 ± 5.54). This study found no correlation between the time from the appearance of the first symptoms to diagnosis and the respondents’ place of residence. The occurrence of menstrual pain was declared by 62% of respondents. Pain complaints ranged from 0 to 10 points, with an average pain score of 4 points (4.07 ± 2.38). Women without children were significantly more likely to visit the center for endometriosis and other conditions, while women with offspring were significantly more likely to report uterine myomas, incontinence, and lowering and prolapse of the reproductive organ (*χ*^2^ = 85.18; *p* < 0.001). Women who had experienced natural childbirth were significantly more likely to report incontinence and lowering and prolapse of the reproductive organ, whereas women who had not undergone this type of childbirth were significantly more likely to indicate endometriosis and other conditions (*χ*^2^ = 64.45; *df* = 5; *p* < 0.001), as shown in Table 4.

The impact of the disease on the material situation was declared as very small by 35.5% of the women surveyed, small by 18%, average by 24%, large by 15.5%, and very large by 7%.

### 3.2. Quality of Life

The assessment of well-being before and after the procedure is shown in Table 5.

The assessment of limitations before and after surgery is shown in Table 6.

This study demonstrated a significant reduction in the level of limitations across all aspects of quality of life (Table 7).

**Figure 1:**
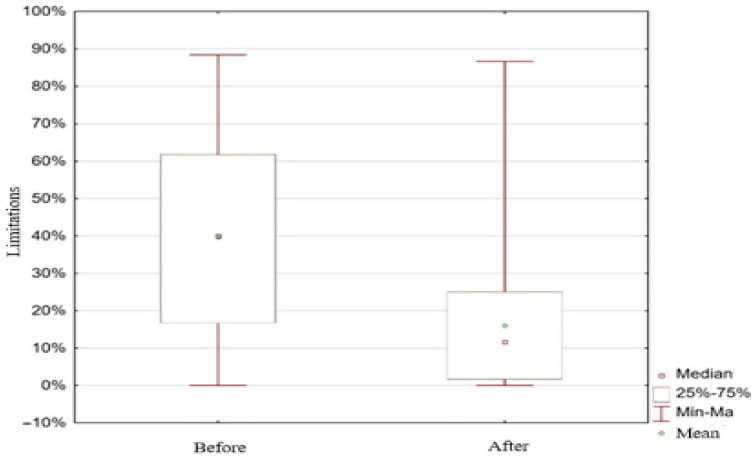
Limitations before and after the procedure.

The level of limitations before surgery ranged from 0% to 88%, while after surgery, it ranged from 0% to 87%. The average level of limitations before surgery among female respondents was 40% (40 ± 25), which was significantly higher (*N* = 180; *z* = 11.24; *p* < 0.001) compared to the average level after surgery, which was 12% (16 ± 17) (Figure 1).

**Figure 2:**
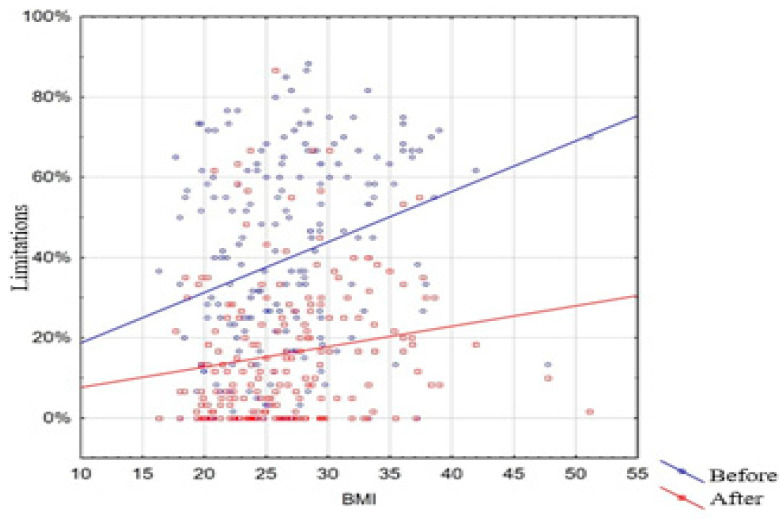
BMI vs. limitations before and after the procedure.

This study observed that a higher BMI was associated with higher levels of limitations both before (*r* = 0.31; *p* < 0.001) and after the procedure (*r* = 0.24; *p* < 0.001) (Figure 2).

In this study, a significant relationship was observed between the age of the respondents and the change in the level of limitations after the procedure concerning most of the aspects studied (Table 8). Women between 36 and 50 years of age demonstrated significantly greater improvement in professional work compared to women over 51 years of age (*z* = 3.15; *p* = 0.005). Women over 51 were characterized by significantly greater improvement in social life compared to women under 35 (*z* = 3.62; *p* < 0.001) and those aged 36–50 (*z* = 3.82; *p* < 0.001). Additionally, women over 51 exhibited significantly greater improvement in sports participation compared to women under 35 (*z* = 2.75; *p* = 0.018). They also showed significantly greater improvement in enjoying their hobbies compared to women under 35 (*z* = 3.51; *p* = 0.001) and those between 36 and 50 (*z* = 2.63; *p* = 0.03). Conversely, women under 35 demonstrated significantly greater improvement in having children compared to women over 51 (*z* = 3.05; *p* = 0.007). Women over 51 exhibited significantly greater improvement in dressing in their favorite way compared to women under 35 (*z* = 5.31; *p* < 0.001) and those aged 36 to 50 (*z* = 3.6; *p* < 0.001). Furthermore, women over 51 years of age showed significantly greater improvement in using public transportation compared to women under 35 (*z* = 6.14; *p* < 0.001) and those aged 36–50 (*z* = 4.1; *p* < 0.001). Women under 35 showed less improvement compared to women aged 36–50 (*z* = 2.44; *p* = 0.044). Women over 51 were characterized by significantly greater improvement in driving compared to women under 35 (*z* = 2.75; *p* = 0.018). They also exhibited significantly greater improvement in going out shopping compared to women under 35 (*z* = 5.89; *p* < 0.001) and those aged 36–50 (*z* = 4.18; *p* < 0.001). Moreover, women over 51 displayed significantly greater improvement in going for longer walks compared to women under 35 (*z* = 5.35; *p* < 0.001) and those aged 36–50 (*z* = 3.28; *p* = 0.003). They were characterized by significantly greater improvement in maintaining weight within the normal range compared to women under 35 (*z* = 4.01; *p* < 0.001) and those aged 36–50 (*z* = 3.68; *p* < 0.001). Women over 51 years of age also demonstrated significantly greater improvement in realizing previously intended plans compared to women under 35 (*z* = 4.08; *p* < 0.001) and those aged 36–50 (*z* = 3.17; *p* = 0.005). Finally, women over 51 exhibited significantly greater improvement in controlling daily expenses compared to women under 35 (*z* = 3.38; *p* = 0.002) and those aged 36–50 (*z* = 3.1; *p* = 0.006).

This study found no relationship between place of residence and the extent of pain and limitation reduction after surgery. Married women demonstrated greater improvement in sexual intercourse and having children, but significantly less improvement in social life, enjoying their hobbies, dressing in their favorite way, using public transportation, going shopping, going for longer walks, and controlling daily expenses (Table 9).

In this study, it was observed that economically active women demonstrated greater improvement in professional work but significantly less improvement in social life, enjoying their hobbies, dressing in their favorite way, using public transportation, going shopping, going for longer walks, maintaining body weight within the normal range, carrying out previously intended plans, and controlling daily expenses (Table 10).

In this study, a significant relationship was observed between the respondents’ weight and the change in the level of limitations after the procedure in most of the aspects studied (Table 11).

Women of normal body weight experienced less improvement in the aspect of social life compared to women who were overweight (*z* = 2.78; *p* = 0.016) and obese (*z* = 3.54; *p* = 0.001). They also experienced less improvement in the enjoyment of hobbies compared to women with obesity (*z* = 2.89; *p* = 0.01). Regarding dressing in their favorite way, normal-weight women showed less improvement compared to both overweight women (*z* = 2.78; *p* = 0.016) and obese women (*z* = 4.94; *p* < 0.001), with overweight women showing less improvement compared to obese women (*z* = 2.44; *p* = 0.045). Normal-weight women also showed less improvement in using public transportation compared to overweight (*z* = 3.93; *p* < 0.001) and obese women (*z* = 4.97; *p* < 0.001). In the aspect of driving, they showed less improvement compared to overweight women (*z* = 2.69; *p* = 0.02). Similarly, they experienced less improvement in going out shopping compared to overweight (*z* = 3.27; *p* = 0.003) and obese women (*z* = 4.08; *p* < 0.001) and in taking longer walks compared to women with obesity (*z* = 3.69; *p* < 0.001). Furthermore, normal-weight women showed less improvement in maintaining weight within the normal range compared to overweight (*z* = 2.86; *p* = 0.01) and obese women (*z* = 4.26; *p* < 0.001) and in achieving previously intended plans compared to women with obesity (*z* = 3.15; *p* = 0.005). They also experienced less improvement in controlling daily expenses compared to women with obesity (*z* = 3.32; *p* = 0.003).

In this study, a significant relationship was observed between the type of condition of the respondents and the change in the level of limitations after surgery with respect to most of the aspects studied (Table 12).

Women with uterine myomas experienced less improvement in social life compared to women with urinary incontinence (*z* = 3.87; *p* < 0.001) and those with lowering or prolapse of the reproductive organ (*z* = 2.72; *p* = 0.04). Similarly, women with uterine myomas showed less improvement in dressing in their favorite way compared to women with urinary incontinence (*z* = 3.85; *p* < 0.001) and those with lowering or prolapse of the reproductive organ (*z* = 2.8; *p* = 0.03). In the aspect of using public transportation, women with uterine myomas exhibited less improvement compared to women with urinary incontinence (*z* = 4.15; *p* < 0.001) and those with lowering or prolapse of the reproductive organ (*z* = 3.08; *p* = 0.01). Likewise, women with uterine myomas showed less improvement in going shopping compared to women with urinary incontinence (*z* = 4.25; *p* < 0.001) and those with lowering or prolapse of the reproductive organ (*z* = 3.35; *p* = 0.005). In the aspect of going for longer walks, women with uterine myomas experienced less improvement compared to women with urinary incontinence (*z* = 4.43; *p* < 0.001) and those with lowering or prolapse of the reproductive organ (*z* = 3.05; *p* = 0.01). Furthermore, women with uterine myomas showed less improvement in maintaining weight within the normal range compared to women with urinary incontinence (*z* = 2.9; *p* = 0.02). Women with uterine myomas also experienced less improvement in achieving previously intended plans compared to women with incontinence (*z* = 2.84; *p* = 0.03). In contrast, women with urinary incontinence showed greater improvement in controlling daily expenses compared to women with uterine myomas (*z* = 3.93; *p* < 0.001) and those with lowering or prolapse of the reproductive organ (*z* = 3.07; *p* = 0.01).

### 3.3. Self-Assessment of Quality of Life

Self-assessment of daily functioning after surgery was rated as average by 6.5%, good by 37.2%, and very good by 56.3% of female respondents. Improvement in daily functioning after surgery was reported by 184 respondents (92.0%), while 3 women indicated no improvement (1.5%), and 13 women were unsure whether their daily functioning had improved after surgery (6.5%). We observed that the current quality of life had significantly improved compared to assessments of quality of life before the disease (*N* = 113; *z* = 5.65; *p* < 0.001) and after diagnosis (*N* = 144; *z* = 9.68; *p* < 0.001). Improvement in quality of life after surgery was reported by 90% of respondents, while 2.5% of women indicated no improvement, and 7.5% of women were unsure whether their quality of life had improved after surgery. Results from the self-assessment of quality of life are shown in Table 13.

In this study, it was observed that normal-weight women had significantly higher quality of life scores before the disease, while obese women had significantly lower quality of life scores after surgery (Table 14).

## 4. Discussion

Quality of life in medicine has long been a significant concern, primarily pondered by physicians and all other medical personnel. This is particularly relevant in areas of medicine where both the conditions being treated are characterized by a high degree of pain experienced by patients, and the treatments themselves can contribute to a temporary reduction in the quality and efficiency of patients’ lives [13]. Such a situation also applies to women struggling with various gynecological diseases, including the respondents in this study.

Many studies utilize validated measures to assess changes in quality of life after surgery, such as the 36-item Short Form Health Survey (SF-36) [14], the EuroQol Health Survey (EQ-5D) [15], or the Veterans RAND 12-item Health Survey (VR-12) [16]. Another method involves simply asking patients how their quality of life changed after surgery, often referred to as a “global assessment” measure [17,18]. Following surgery and anesthesia, patients can be classified based on their QoR-15 score [19]. Both methods aim to measure the same construct, namely health-related quality of life. Validated measures are more objective as they inquire about concrete factors contributing to quality of life; however, they can be time-consuming to administer. In contrast, the global measure is quicker to administer but more subjective [20]. In the study conducted, we used our own questionnaire, adapted to the clinical situation of the patients. This approach provided an opportunity to gain insights into their overall health status, living conditions, and recovery process.

Women of all ages are treated at the Minimally Invasive Pelvic Surgery Center at the Gynecological and Obstetrics Clinical Hospital in Poznań, Poland. For the most part, these age groups align with those noted by other researchers: women over the age of 50 most often seek treatment for urinary incontinence, while younger women typically present with endometriosis and/or uterine myomas [21]. Importantly, occupationally active women were significantly more likely to seek surgery for the removal of uterine myomas or endometriosis [22]. Women with endometriomas reported significantly more problems in the areas of “paid work” (*p* < 0.001), “housework” (*p* < 0.001), social life (*p* < 0.001), and sexual life (*p* < 0.001), as well as problems in continuing hobbies (*p* < 0.001) and spending leisure time (*p* < 0.001) [23]. Factors such as being professionally inactive [22], patient age (*p* = 0.0001), body mass index (BMI) (*p* = 0.0001), and the surgical procedure for the removal of the uterus via laparotomy (*p* = 0.0001) exert the greatest effect on the occurrence of stress urinary incontinence (SUI) in premenopausal and postmenopausal women [24].

This study found that the majority of the examined women who underwent minimally invasive surgical techniques (laparoscopic) rated their quality of life as good. This result is mainly attributed to women suffering from various gynecological conditions for an extended period, after receiving appropriate treatment and undergoing the procedure, experiencing significant improvement in their health [21,25,26]. First and foremost, this improvement is related to the pain experienced by the patients, as well as various limitations, such as an inability to carry out heavier shopping or participate in sports [21].

Accordingly, the surgery the subjects underwent positively impacted their lives, primarily improving their quality of life. The most important positive aspect of the surgery/procedure is that most of the participants stopped experiencing pain, allowing them to resume many of their normal life activities. This, in turn, led the women to feel no regret about their decision to undergo the procedure.

The type of surgical method also affects the length of hospitalization; the longest average hospitalization time was observed in the group of women operated on via laparotomy, with an average of 5.29 days. In contrast, the hospitalization time was more than half as short in the group of women operated on laparoscopically, averaging 2.45 days [21]. Recovery times lengthened with increasing levels of physical burden, as well as with the invasiveness of the surgery [27]. Thus, the type of surgery performed and the shorter hospitalization time associated with the laparoscopic technique realistically affect the better quality of life of patients after the procedure [28]. Another important element of quality of life for women after surgery is the need for convalescence and the various inconveniences associated with it. Psychological factors, demographic factors, and perioperative outcomes are important predictors of recovery quality and convalescence duration. Increased recovery quality is associated with a shorter convalescence period [29,30]. When a woman requires surgery, she primarily considers the need for a long hospital stay associated with both preparation for the procedure and recovery after the operation. Despite the fact that this time is significantly reduced today and that surgical treatments are becoming less invasive, it is important to note that many patients still experience a kind of “suspension” in their lives. Some patients are on long-term sick leave [31], and the awareness of being unable to carry out daily activities—particularly those related to work and family life—can significantly affect how a woman perceives her situation and assesses her quality of life. The level of their quality of life has improved or may improve after the procedure, which is clearly related to the relief experienced by the patients [32].

Patients who underwent surgery using minimally invasive techniques rated their quality of life as good. The treatment positively impacted their daily functioning. In most cases, the women reported a significant reduction in pain and the ability to engage in activities they had given up on due to their condition. The most common complaints reported were widespread discomfort and the need for regular doctor visits for check-ups or treatments. The respondents did not regret their decision to undergo the procedure. This is primarily due to the significant improvement in their quality of life and their ability to return to daily activities. The level of their quality of life improved or may improve after the procedure, which is clearly related to the relief they experienced.

Study limitations: The questionnaire was developed specifically for our study population (the Minimally Invasive Pelvic Surgery Center at Heliodor Święcicki Gynecological and Obstetrics Clinical Hospital of the Karol Marcinkowski Medical University in Poznań, Poland); the lack of validation against standard instruments (SF-36, EQ-5D) limits the comparability of results and the generalizability of conclusions, calling for cautious interpretation of the findings. Consequently, the model’s results or predictions may be difficult to trust or explain, as the apparent relationships may not hold beyond the specific dataset used. However, the use of questionnaires that have not been validated is not a weakness of our work; such are the demands of our times of living online and on social media.

## 5. Conclusions

1. Women with uterine fibroids experienced less improvement in QoL than those treated for prolapse or incontinence.
2. Psychological factors, such as anxiety and long-term distress, play an important role in both pre- and postoperative well-being.
3. The use of a customized, non-validated questionnaire enabled a context-specific evaluation of patients’ recovery experiences.
4. Optimizing postoperative quality of life requires consideration of both the surgical method and individual psychosocial circumstances.

## Figures and Tables

**Table 1:** Characteristics of the study group.

Age	<25	2.5%
	26–35	22%
	36–50	36.5%
	51–65	20.5%
	>65	18.5%
Place of residence	Village	23.5%
	City with population < 10 thousand	27.5%
	City with population of 10–500 thousand	21.5%
	City with population > 500 thousand	27.5%
Marital status	Single	6.5%
	Partnership	10.5%
	Married	55%
	Divorced	11.5%
	Widowed	16.5%
Occupational activity	Studying	0.5%
	Studying and working	6.5%
	Working	59.5%
	Sick leave	9%
	Pensioner/Retiree	24.5%
BMI	Underweight—BMI: <18.5 kg/m^2^	3.5%
	Normal body weight—BMI: 18.5–24.9 kg/m^2^	40%
	Overweight—BMI: 25–29.9 kg/m^2^	34.5%
	Obesity—BMI: ≥30 kg/m^2^	22%

**Table 2:** Anthropometric measurements.

	M ± SD	Min–Max	Me [Q1–Q3]
Height (cm)	165.09 ± 5.52	150–179	165 [160–170]
Body weight (kg)	72.5 ± 15.27	48–130	70 [60–82]
BMI	26.64 ± 5.67	16.33–51.11	26.03 [22.41–29.4]

**Table 3:** Age, occupational activity, and body weight vs. type of condition.

	Age		
	<35 Years	36–50 Years	>51 Years
Uterine myomas	3 (6.12%)	36 (49.32%)	8 (10.26%)
Urinary incontinence	0 (0%)	7 (9.59%)	31 (39.74%)
Lowering of reproductive organ	2 (4.08%)	3 (4.11%)	11 (14.1%)
Prolapse of reproductive organ	0 (0%)	5 (6.85%)	24 (30.77%)
Endometriosis	15 (30.61%)	6 (8.22%)	1 (1.28%)
Other	29 (59.18%)	16 (21.92%)	3 (3.85%)
Chi^2^ NW	*χ*^2^ = 163.98; *df* = 10; *p* < 0.001		
	**Body Weight Assessment**		
	**Normal**	**Overweight**	**Obesity**
Uterine myomas	22 (27.5%)	17 (24.64%)	7 (15.91%)
Urinary incontinence	5 (6.25%)	14 (20.29%)	19 (43.18%)
Lowering of reproductive organ	3 (3.75%)	6 (8.7%)	7 (15.91%)
Prolapse of reproductive organ	4 (5%)	17 (24.64%)	8 (18.18%)
Endometriosis	11 (13.75%)	8 (11.59%)	0 (0%)
Other	35 (43.75%)	7 (10.14%)	3 (6.82%)
Chi^2^ NW	*χ*^2^ = 71.97; *df* = 10; *p* < 0.001		

**Table 4:** Birth rate and type of delivery vs. type of condition.

	Birth Rate		
	Childless	Primiparous	Multiparous
Uterine myomas	4 (9.76%)	18 (28.57%)	25 (26.04%)
Urinary incontinence	0 (0%)	14 (22.22%)	24 (25%)
Lowering of reproductive organ	1 (2.44%)	5 (7.94%)	10 (10.42%)
Prolapse of reproductive organ	0 (0%)	5 (7.94%)	24 (25%)
Endometriosis	11 (26.83%)	7 (11.11%)	4 (4.17%)
Other	25 (60.98%)	14 (22.22%)	9 (9.38%)
Chi^2^ NW	*χ*^2^ = 85.18; *df* = 10; *p* < 0.001		
		**Natural Delivery**	
		**Yes**	**No**
Uterine myomas		28 (25%)	19 (21.59%)
Urinary incontinence		28 (25%)	10 (11.36%)
Lowering of reproductive organ		14 (12.5%)	2 (2.27%)
Prolapse of reproductive organ		26 (23.21%)	3 (3.41%)
Endometriosis		2 (1.79%)	20 (22.73%)
Other		14 (12.5%)	34 (38.64%)
Chi^2^ NW		*χ*^2^ = 64.45; *df* = 5; *p* < 0.001	

**Table 5:** Assessment of well-being before and after the procedure.

Assessment of Well-Being Before the Procedure		Assessment of Well-Being After the Procedure	
Poor	3%	Poor	0.5%
Moderate	25.5%	Moderate	3.5%
Good	62.5%	Good	40.5%
Very good	9%	Very good	55.5%

**Table 6:** Limitations before and after surgery.

	Surgery						Wilcoxon Test		
	Before			After			N	Z	*p*
	M ± SD	Min–Max	Me [Q1–Q3]	M ± SD	Min–Max	Me [Q1–Q3]			
I ^a^	1.43 ± 1.46	0–4	1 [0–3]	0.59 ± 0.9	0–4	0 [0–1]	105	8.39	<0.001
II ^b^	2.03 ± 1.51	0–4	2 [0–3]	0.8 ± 0.95	0–4	1 [0–1]	134	9.50	<0.001
III ^c^	2.22 ± 1.54	0–4	3 [0.5–4]	0.89 ± 1.06	0–4	1 [0–1]	145	9.80	<0.001
IV ^d^	1.84 ± 1.51	0–4	2 [0–3]	0.74 ± 1.02	0–4	0 [0–1]	121	8.59	<0.001
V ^e^	1.94 ± 1.59	0–4	2 [0–3]	0.85 ± 1.02	0–4	1 [0–1]	120	8.68	<0.001
VI ^f^	0.86 ± 1.5	0–4	0 [0–1]	0.45 ± 1.01	0–4	0 [0–0]	55	4.57	<0.001
VII ^g^	1.82 ± 1.63	0–4	2 [0–3]	0.64 ± 0.95	0–4	0 [0–1]	111	8.86	<0.001
VIII ^h^	1.62 ± 1.68	0–4	1 [0–3]	0.62 ± 0.94	0–4	0 [0–1]	104	8.36	<0.001
IX ^i^	0.86 ± 1.25	0–4	0 [0–2]	0.34 ± 0.75	0–4	0 [0–0]	68	5.90	<0.001
X ^j^	0.75 ± 1.35	0–4	0 [0–1]	0.33 ± 0.77	0–4	0 [0–0]	54	4.88	<0.001
XI ^k^	1.64 ± 1.59	0–4	1 [0–3]	0.64 ± 0.89	0–4	0 [0–1]	117	8.25	<0.001
XII ^l^	2.01 ± 1.6	0–4	2 [0–4]	0.72 ± 0.92	0–4	0 [0–1]	126	9.51	<0.001
XIII ^m^	1.88 ± 1.6	0–4	2 [0–3]	0.79 ± 1.01	0–4	0.5 [0–1]	120	8.87	<0.001
XIV ^n^	1.94 ± 1.62	0–4	2 [0–4]	0.78 ± 1.09	0–4	0 [0–1]	121	9.07	<0.001
XV ^o^	0.99 ± 1.43	0–4	0 [0–2]	0.48 ± 0.87	0–4	0 [0–1]	76	5.64	<0.001

^a^ Working life, ^b^ Social life, ^c^ Playing sports, ^d^ Sexual intercourse, ^e^ Enjoying hobbies, ^f^ Having children, ^g^ Dressing in favorite way, ^h^ Using public transportation, ^i^ Driving a car, ^j^ Having a partner, ^k^ Going out shopping, ^l^ Going for a long walk, ^m^ Keeping body weight within the normal range, ^n^ Carrying out previously intended plans, ^o^ Controlling daily expenses.

**Table 7:** Extent of reduction in limitations after surgery.

	M ± SD	Min–Max	Me [Q1–Q3]
I ^a^	−0.85 ± 1.06	−4–2	−0.5 [−2–0]
II ^b^	−1.23 ± 1.23	−4–4	−1 [−2–0]
III ^c^	−1.34 ± 1.28	−4–2	−2 [−2–0]
IV ^d^	−1.1 ± 1.36	−4–4	−1 [−2–0]
V ^e^	−1.09 ± 1.28	−4–3	−1 [−2–0]
VI ^f^	−0.42 ± 1.19	−4–3	0 [0–0]
VII ^g^	−1.18 ± 1.34	−4–2	−1 [0–2]
VIII ^h^	−1 ± 1.26	−4–2	0 [−2–0]
IX ^i^	−0.52 ± 1.09	−4–4	0 [−1–0]
X ^j^	−0.42 ± 1.1	−4–3	0 [0–0]
XI ^k^	−1 ± 1.34	−4–3	−1 [−2–0]
XII ^l^	−1.29 ± 1.29	−4–1	−1 [−2–0]
XIII ^m^	−1.09 ± 1.27	−4–4	−1 [−2–0]
XIV ^n^	−1.16 ± 1.32	−4–2	−1 [−2–0]
XV ^o^	−0.51 ± 1.17	−4–4	0 [−1–0]

^a^ Working life, ^b^ Social life, ^c^ Playing sports, ^d^ Sexual intercourse, ^e^ Enjoying hobbies, ^f^ Having children, ^g^ Dressing in favorite way, ^h^ Using public transportation, ^i^ Driving a car, ^j^ Having a partner, ^k^ Going out shopping, ^l^ Going for a long walk, ^m^ Keeping body weight within the normal range, ^n^ Carrying out previously intended plans, ^o^ Controlling daily expenses.

**Table 8:** Age and the extent of pain and limitation reduction after surgery.

	Age			H	*p*
	<35 Years	36–50 Years	>51 Years		
I ^a^	−1 [−2–0]	−1 [−2–0]	0 [−1–0]	11.43	0.003
II ^b^	0 [−2–0]	−1 [−2–0]	−2 [−3–−1]	20.81	<0.001
III ^c^	−1 [−2–0]	−2 [−2–0]	−2 [−3–0]	8.62	0.01
IV ^d^	−1 [−2–0]	−2 [−2–0]	0 [−2–0]	2.46	0.29
V ^e^	0 [−1–0]	−1 [−2–0]	−2 [−2–0]	15.28	<0.001
VI ^f^	0 [−2–0]	0 [0–0]	0 [0–0]	15.16	<0.001
VII ^g^	0 [0–0]	0 [−2–0]	−2 [−3–−1]	33.79	<0.001
VIII ^h^	0 [0–0]	0 [−2–0]	−2 [−3–−1]	45.91	<0.001
IX ^i^	0 [0–0]	0 [−1–0]	0 [−2–0]	11.40	0.003
X ^j^	0 [0–0]	0 [−1–0]	0 [0–0]	1.90	0.39
XI ^k^	0 [0–0]	0 [−2–0]	−2 [−3–−1]	41.57	<0.001
XII ^l^	0 [−1–0]	−1 [−2–0]	−2 [−3–−1]	32.16	<0.001
XIII ^m^	0 [−1–0]	0 [−2–0]	−2 [−3–−1]	22.62	<0.001
XIV ^n^	0 [−1–0]	0 [−2–0]	−2 [−3–−1]	20.70	<0.001
XV ^o^	0 [0–0]	0 [0–0]	−1 [−2–0]	19.41	<0.001
XVI ^p^	−2 [−4–0]	−2 [−3–0]	−2 [−3–−1]	0.23	0.89

^a^ Working life, ^b^ Social life, ^c^ Playing sports, ^d^ Sexual intercourse, ^e^ Enjoying hobbies, ^f^ Having children, ^g^ Dressing in favorite way, ^h^ Using public transportation, ^i^ Driving a car, ^j^ Having a partner, ^k^ Going out shopping, ^l^ Going for a long walk, ^m^ Keeping body weight within the normal range, ^n^ Carrying out previously intended plans, ^o^ Controlling daily expenses, ^p^ Pain.

**Table 9:** Marital status and the extent of pain and limitation reduction after surgery.

	Marital Status		U	*p*
	Married	Other		
I ^a^	−1 [−2–0]	0 [−2–0]	4305	0.09
II ^b^	−1 [−2–0]	−2 [−2–0]	3941	0.01
III ^c^	−2 [−2–0]	−1.5 [−2–0]	4882	0.86
IV ^d^	−1.5 [−2–0]	0 [−2–0]	3781.5	0.003
V ^e^	0 [−2–0]	−1.5 [−2–0]	4058.5	0.02
VI ^f^	0 [−1–0]	0 [0–0]	4220	0.02
VII ^g^	0 [−2–0]	−1 [−3–0]	4077	0.02
VIII ^h^	0 [−2–0]	−1 [−2–0]	3749.5	0.002
IX ^i^	0 [−1–0]	0 [−1–0]	4909.5	0.91
X ^j^	0 [−1–0]	0 [0–0]	4396.5	0.08
XI ^k^	0 [−2–0]	−1 [−3–0]	4050	0.02
XII ^l^	−1 [−2–0]	−1.5 [−3–0]	4156	0.04
XIII ^m^	−1 [−2–0]	−1 [−3–0]	4249	0.07
XIV ^n^	−1 [−2–0]	−1 [−3–0]	4394	0.16
XV ^o^	0 [−1–0]	0 [−2–0]	4106.5	0.02
XVI ^p^	−2 [−3–0]	−2 [−3–−1]	4281.5	0.10

^a^ Working life, ^b^ Social life, ^c^ Playing sports, ^d^ Sexual intercourse, ^e^ Enjoying hobbies, ^f^ Having children, ^g^ Dressing in favorite way, ^h^ Using public transportation, ^i^ Driving a car, ^j^ Having a partner, ^k^ Going out shopping, ^l^ Going for a long walk, ^m^ Keeping body weight within the normal range, ^n^ Carrying out previously intended plans, ^o^ Controlling daily expenses, ^p^ Pain.

**Table 10:** Occupational activity and the extent of pain and limitation reduction after surgery.

	Occupational Activity		U	*p*
	Yes	No		
I ^a^	−1 [−2–0]	0 [−1–0]	2955.5	<0.001
II ^b^	−1 [−2–0]	−2 [−2–−1]	3263	0.001
III ^c^	−2 [−2–0]	−2 [−2.5–0]	4129.5	0.34
IV ^d^	−1 [−2–0]	0 [−2–0]	3941.5	0.14
V ^e^	0 [−2–0]	−2 [−2–0]	3552	0.01
VI ^f^	0 [−1–0]	0 [0–0]	3912.5	0.06
VII ^g^	0 [−2–0]	−2 [−3–0]	3402	0.003
VIII ^h^	0 [−2–0]	−1 [−2.5–0]	3358.5	0.002
IX ^i^	0 [−1–0]	0 [−1–0]	4485.5	1.00
X ^j^	0 [0–0]	0 [0–0]	4318	0.58
XI ^k^	0 [−2–0]	−1 [−3–0]	3324	0.002
XII ^l^	−1 [−2–0]	−2 [−3–−1]	3136	<0.001
XIII ^m^	−1 [−2–0]	−2 [−3–0]	3447	0.005
XIV ^n^	0 [−2–0]	−1.5 [−3–−0.5]	3219.5	<0.001
XV ^o^	0 [0–0]	−1 [−2–0]	3169	<0.001
XVI ^p^	−2 [−3–0]	−2 [−3–−1]	4359.5	0.74

^a^ Working life, ^b^ Social life, ^c^ Playing sports, ^d^ Sexual intercourse, ^e^ Enjoying hobbies, ^f^ Having children, ^g^ Dressing in favorite way, ^h^ Using public transportation, ^i^ Driving a car, ^j^ Having a partner, ^k^ Going out shopping, ^l^ Going for a long walk, ^m^ Keeping body weight within the normal range, ^n^ Carrying out previously intended plans, ^o^ Controlling daily expenses, ^p^ Pain.

**Table 11:** Body weight assessment and the extent of pain and limitation reduction after surgery.

	Body Weight Assessment			H	*p*
	Normal	Overweight	Obesity		
I ^a^	−1 [−2–0]	0 [−1–0]	0 [−2–0]	2.69	0.26
II ^b^	−1 [−2–0]	−2 [−2–0]	−2 [−2.5–−0.5]	15.78	<0.001
III ^c^	−1 [−2–0]	−2 [−2–0]	−2 [−2.5–0]	1.43	0.49
IV ^d^	−1 [−2–0]	−1 [−2–0]	0 [−2–0]	1.05	0.59
V ^e^	0 [−2–0]	−1 [−2–0]	−2 [−2–0]	9.26	0.01
VI ^f^	0 [−1–0]	0 [0–0]	0 [0–0]	7.71	0.02
VII ^g^	0 [−1–0]	−1 [−2–0]	−2 [−3–−1.5]	27.94	<0.001
VIII ^h^	0 [−1–0]	−1 [−2–0]	−2 [−3–−0.5]	32.85	<0.001
IX ^i^	0 [0–0]	0 [−1–0]	0 [−2–0]	10.54	0.005
X ^j^	0 [0–0]	0 [−1–0]	0 [0–0]	2.91	0.23
XI ^k^	0 [−1–0]	−1 [−2–0]	−2 [−3–0]	21.49	<0.001
XII ^l^	0 [−2–0]	−1 [−2–0]	−2 [−3–−1]	15.48	<0.001
XIII ^m^	0 [−1–0]	−1 [−2–0]	−2 [−3–−1]	21.48	<0.001
XIV ^n^	0 [−1.5–0]	−1 [−2–0]	−2 [−3–0]	12.06	0.002
XV ^o^	0 [0–0]	0 [−1–0]	−1 [−2–0]	15.48	<0.001
XVI ^p^	−2 [−3–0]	−2 [−3–0]	−2 [−3–−1]	0.03	0.98

^a^ Working life, ^b^ Social life, ^c^ Playing sports, ^d^ Sexual intercourse, ^e^ Enjoying hobbies, ^f^ Having children, ^g^ Dressing in favorite way, ^h^ Using public transportation, ^i^ Driving a car, ^j^ Having a partner, ^k^ Going out shopping, ^l^ Going for a long walk, ^m^ Keeping body weight within the normal range, ^n^ Carrying out previously intended plans, ^o^ Controlling daily expenses, ^p^ Pain.

**Table 12:** Kind of condition and the extent of pain and limitation reduction after surgery.

	Kind of Condition				H	*p*
	Uterine Myomas	Urinary Incontinence	Lowering/Prolapse of Reproductive Organ	Other		
I ^a^	−1 [−2–0]	0 [−2–0]	0 [−1–0]	−1 [−2–0]	4.17	0.24
II ^b^	−1 [−2–0]	−2 [−3–−2]	−2 [−2–−1]	0 [−1–0]	32.19	<0.001
III ^c^	−2 [−2–0]	−2 [−3–0]	−2 [−2–−1]	−1 [−2–0]	6.77	0.08
IV ^d^	−2 [−2–0]	0 [−2–0]	−1 [−2–0]	−1 [−2–0]	6.50	0.09
V ^e^	0 [−2–0]	−2 [−3–0]	−2 [−2–0]	0 [−2–0]	13.12	0.004
VI ^f^	0 [0–0]	0 [0–0]	0 [0–0]	0 [−2–0]	10.91	0.01
VII ^g^	0 [−2–0]	−2.5 [−3–−1]	−2 [−2–−1]	0 [−1–0]	43.22	<0.001
VIII ^h^	0 [−2–0]	−2 [−3–−1]	−2 [−2–−1]	0 [0–0]	56.70	<0.001
IX ^i^	0 [−1–0]	0 [−2–0]	0 [−2–0]	0 [0–0]	14.33	0.003
X ^j^	0 [−1–0]	0 [0–0]	0 [−1–0]	0 [0–0]	1.05	0.79
XI ^k^	0 [−2–0]	−2 [−3–−1]	−2 [−3–−1]	0 [−1–0]	49.92	<0.001
XII ^l^	−1 [−2–0]	−2.5 [−3–−2]	−2 [−3–−1]	0 [−1–0]	43.49	<0.001
XIII ^m^	0 [−2–0]	−2 [−3–0]	−2 [−2–−1]	0 [−1–0]	22.68	<0.001
XIV ^n^	−1 [−2–0]	−2 [−3–−1]	−1 [−2–−1]	0 [−1–0]	21.25	<0.001
XV ^o^	0 [0–0]	−1.5 [−2–0]	0 [−1–0]	0 [0–0]	28.91	<0.001
XVI ^p^	−2 [−3–−1]	−2 [−2–0]	−2 [−3–−1]	−2 [−4–0]	3.38	0.34

^a^ Working life, ^b^ Social life, ^c^ Playing sports, ^d^ Sexual intercourse, ^e^ Enjoying hobbies, ^f^ Having children, ^g^ Dressing in favorite way, ^h^ Using public transportation, ^i^ Driving a car, ^j^ Having a partner, ^k^ Going out shopping, ^l^ Going for a long walk, ^m^ Keeping body weight within the normal range, ^n^ Carrying out previously intended plans, ^o^ Controlling daily expenses, ^p^ Pain.

**Table 13:** Self-assessment of quality of life.

Self-Assessment of Quality of Life	Before Diagnosis	After Diagnosis	Currently
Poor	4%	7%	5%
Moderate	14.5%	37%	40.5%
Good	52.5%	50%	54.5%
Very good	29%	6%	0%

**Table 14:** Body weight assessment and quality of life.

Quality of Life		Body Weight Assessment		
		Normal	Overweight	Obesity
Before diagnosis	Poor	1 (1.25%)	7 (10.14%)	0 (0%)
	Moderate	12 (15%)	10 (14.49%)	6 (13.64%)
	Good	33 (41.25%)	40 (57.97%)	28 (63.64%)
	Very good	34 (42.5%)	12 (17.39%)	10 (22.73%)
	Chi^2^ NW	*χ*^2^ = 22.39; *df* = 6; *p* = 0.001		
After diagnosis	Poor	4 (5%)	8 (11.59%)	2 (4.55%)
	Moderate	31 (38.75%)	25 (36.23%)	17 (38.64%)
	Good	40 (50%)	32 (46.38%)	24 (54.55%)
	Very good	5 (6.25%)	4 (5.8%)	1 (2.27%)
	Chi^2^ NW	*χ*^2^ = 4.21; *df* = 6; *p* = 0.65		
Currently	Moderate	2 (2.5%)	6 (8.7%)	2 (4.55%)
	Good	30 (37.5%)	19 (27.54%)	31 (70.45%)
	Very good	48 (60%)	44 (63.77%)	11 (25%)
	Chi^2^ NW	*χ*^2^ = 24.34; *df* = 4; *p* < 0.001		

## Data Availability

The data presented in this study are available on request from the corresponding author. The data are not publicly available due to institutional restrictions.

## Abbreviations

The following abbreviations are used in this manuscript:

BMI	Body Mass Index;
CC BY	Creative Commons Attribution;
EQ-5D	EuroQol 5-Dimensions Health Survey;
QoL	Quality of Life;
QoR-15	Quality of Recovery-15 Questionnaire;
SF-36	36-Item Short Form Health Survey;
SUI	Stress Urinary Incontinence;
VR-12	Veterans RAND 12-Item Health Survey.
